# Sustainability and future outlook of the Philippine pig industry

**DOI:** 10.5455/javar.2025.l978

**Published:** 2025-12-25

**Authors:** Md. Sharifuzzaman, Hong-Seok Mun, Eddiemar B. Lagua, Hae-Rang Park, Young-Hwa Kim, Md. Kamrul Hasan, Jin-Gu Kang, Chul-Ju Yang

**Affiliations:** 1 Animal Nutrition and Feed Science Laboratory, Department of Animal Science and Technology, Sunchon National University, Suncheon, Republic of Korea; 2 Department of Animal Science and Veterinary Medicine, Gopalganj Science and Technology University, Gopalganj, Bangladesh; 3 Department of Multimedia Engineering, Sunchon National University, Suncheon, Republic of Korea; 4 Interdisciplinary Program in IT-Bio Convergence System (BK21 Plus), Sunchon National University, Suncheon, Republic of Korea; 5 Interdisciplinary Program in IT-Bio Convergence System (BK21 Plus), Chonnam National University, Gwangju, Republic of Korea; 6 Department of Poultry Science, Sylhet Agricultural University, Sylhet, Bangladesh

**Keywords:** Philippines, Swine, Technology, Pollution, Sustainability

## Abstract

The Philippines relies significantly on swine-related enterprises for food security and economic stability. The sector has faced numerous restraints in recent years and remains considerably distant from reaching the production target for domestic consumption. This study aims to identify the key drivers of the nation’s pork production in recent years, thereby enhancing our understanding of what is needed to make the industry sustainable in the future. A comprehensive review was conducted using keyword-based searches across major databases and official reports (2018–2023) to assess pig production, consumption, technology adoption, and sustainability in the Philippines. The extracted data were analyzed using Pearson correlation analysis in IBM SPSS Statistics 20 to examine the relationships among key production factors. Most of the existing problems identified through this review are somehow related to small-scale operation. Large-scale commercial farms have solutions to many of these issues, and a gradual expansion of their operations is recommended. We observed a powerful negative linear relationship between domestic pork production and pork importation (*r* = −0.949). Pork importation contributes to retail price hike (*r* = 0.948) and is negatively related to consumption (*r* = −0.815), indicating that increasing national production is mandatory for stabilizing the market. A rapid transition to commercial systems is not feasible, as many farmers would be left with no alternatives if the government were to cease support. Consistent guidance, support, and monitoring from the government and other responsible entities can help build awareness, establish cooperative farms, and achieve sustainability.

## Introduction

As a Southeast Asian archipelago with diverse climates and landscapes [1], the Philippines relies heavily on agriculture as a backbone of its economy and a key driver of national development [2,3]. It is essential for maintaining food security and reducing poverty [4-7]. Over the past 8 years, the agriculture sector (encompassing agriculture, forestry, and fisheries) has contributed an average of approximately 9.59% to the Philippines’ Gross Domestic Product, with the latest contribution of 8.6% in 2023 [8]. Agriculture also represents the largest share of the labor force, employing around 24.3% of the labor force from 2016 to 2023 [9]. According to a recent report by the Department of Labor and Employment, the agriculture sector alone employed 11.19 million workers in 2023 [10]. The Philippine animal industry substantially contributes to the agriculture sector, accounting for roughly 18.23% of the gross agricultural output value [11]. The industry had a total production value of 263.4 billion Philippine pesos (PHP) in 2023 [12].

Among livestock enterprises, the swine industry is the most significant contributor [13–16], mirroring trends in other developing nations where it serves as a key economic activity, a pathway out of poverty, and a means of enhancing food security [17-19]. The importance of the sector is understood by the fact that 43% of Filipinos working in agriculture are engaged in swine operations [13]. In addition to employment, the swine business is also linked to the country’s food security, as pork is the primary meat consumed [20-22]. It represented approximately 44.15% of 2023’s meat consumption [23]. The Philippines’ pork consumption exceeds the global average [21,24] and is expected to be as high as 15.14 kg in 2024 [25]. According to the Philippine Statistics Authority (PSA), 62% of households in the Philippines consume pork [24]. They love processed items, including ham, samgyeopsal-gui, tocino, longganisa, galbi-gui, hotdogs, siopao, bacon, lechon, and so on [26,27]. In terms of pork production, the country ranks third in Asia, trailing behind Vietnam and China [28], and holds the 11th position globally [29].

Despite the presence of large-scale swine farms in some parts of the Philippines, backyard hog raising remains the dominant practice in the country [16,20,28,30]. Maintaining adequate nutrition is essential in low- and middle-income countries, such as the Philippines [1,31]. Smallholder farmers contribute 70.6% of the total hog production [32]. Pigs are frequently regarded as companions by growers, with some females even likening them to their kids [18]. Despite its contributions to livelihoods and food security, as well as emotional attachments to the raisers, traditional pig production is characterized by low output standards [33-35]. The scattered nature of backyard production on a large scale poses a challenge for achieving sustainable development [36]. In contrast, commercial swine farms are characterized by better production performance, wider market reach, and higher profitability [17]. To meet the growing demand for pork, a transition from backyard to commercial operations is essential. However, increased production in commercial settings to address hunger and food security comes with higher waste generation, contributing to intensified pollution of water, soil, and air [37-39]. The shift must be accompanied by efforts to address pollution and other challenges through significant infrastructure development, technical assistance, technological advancements, capital investments, and the implementation of contemporary agricultural technologies in the Philippines.

This article aims to examine the factors influencing the advancement and the obstacles hindering the development of pig farming in the Philippines through a comprehensive literature review. Unlike previous reviews, this paper integrates multiple dimensions, including production statistics, marketing trends, technological adoption, environmental challenges, and sustainability prospects to provide a comprehensive assessment of the swine industry. The research questions addressed in this paper are as follows:

1. What factors influence the development of pig farming in the Philippines, and what challenges hinder the transition from backyard to commercial operations?
2. What are the environmental impacts of increased swine production, and how can infrastructural, technical, and financial investments, along with precision farming technologies, mitigate these challenges?
3. How can the Philippine swine industry enhance its resilience to challenges such as disease outbreaks, including African Swine Fever (ASF), while ensuring sustainable growth?

The initial section of the article delineates the contemporary state of swine farming in the Philippines, encompassing production status, rearing practices, product marketing, and associated concerns. The subsequent section presents an overview of innovative technology and methodologies globally in efficient and sustainable pig production. The following sections examine the potential for sustainable swine farming in the country. Ultimately, recommendations are established to provide potential tactics for fortifying the industry while considering sustainable development.

## Methodology

### Article retrieval and selection

A comprehensive investigation was conducted to identify relevant material regarding the current status and future possibilities of pig farming in the Philippines. Due to their wide range of academic pieces in the fields of livestock husbandry and smart agriculture, Google Scholar, ScienceDirect, and Web of Science archives were searched using search items related to the Philippines, swine production, pig husbandry, ecological effects, sustainability, and sophisticated pig raising. To ensure inclusivity and precision, Boolean operators were applied in different combinations. The primary search string included: “Philippines” AND (“swine” OR “pig” OR “hog”) AND (“production” OR “husbandry”) AND (“technology” OR “smart farming” OR “mechanization”) AND (“pollution” OR “environment” OR “sustainability”). Additional searches were conducted using combinations such as “Philippines” AND “swine production” AND “African swine fever”, “Philippines” AND “pork consumption” AND “market trends”, and so on.

Furthermore, to obtain a diverse array of viewpoints and the latest developments, online resources were integrated, including official government documents, policy papers, reports from international initiatives, and credible news outlets. The period from 2018 to 2023 was considered to encompass both pre- and post-ASF outbreak phases. The review included studies that met the following criteria: (a) abstracts focused on challenges and opportunities in pig farming in the Philippines; (b) provided statistics regarding hog and pork production, distribution, and consumption; (c) examined mechanization in precise animal husbandry; and (d) were disseminated in journals with peer review, esteemed web pages, governmental publications, or conference proceedings. All inclusions were restricted to English. A bibliographic management software (Zotero) was used to process citations according to citation style specifications.

### Data acquisition and statistical analysis

To facilitate the recognition of patterns, issues, possibilities, and developing trends, relevant information was extracted from selected literature sources (including charts and text) and systematically formatted in a Microsoft Excel document for tabular and visual illustrations in the article. Pearson correlation analysis was conducted using IBM SPSS Statistics version 20 to examine the relationships among key parameters influencing pork production in the Philippines, including hog population, farmgate and retail price, pork consumption, broiler price, annual family income, and other relevant traits. The correlation results are presented and interpreted in the “Understanding the Recent Trend” section.

## Current Pig Production Statistics

### Production volume and trends

Pork significantly outweighs other livestock and poultry in terms of population and contribution in the Philippines. It was once the most consumed single meat until 2020. Strikes of ASF have shifted the consumption pattern slightly towards chicken meat. The farm animal productivity situation in the Philippines from 2018 to 2023 is presented in Table 1.

**Table 1.** Livestock production trend of the Philippines from 2018 to 2023.

Year	Population (Millions)				Volume of Production (Liveweight, MMT)					References
	Pig	Cattle	Carabao	Goat	Pork	Chicken	Beef	Carabao	Goat	
2018	12.60	2.55	2.88	3.72	2.32	1.84	0.26	0.14	0.08	[40]
2019	12.71	2.54	2.87	3.76	2.30	1.93	0.26	0.14	0.08	[40]
2020	12.80	2.54	2.87	3.81	2.14	1.81	0.23	0.12	0.07	[41]
2021	9.94	2.61	2.85	3.87	1.70	1.74	0.24	0.13	0.07	[41]
2022	9.43	2.58	2.77	3.91	1.74	1.87	0.24	0.13	0.07	[41]
2023	9.77	2.58	2.78	3.90	1.79	1.95	0.23	0.13	0.07	[41]

The Philippines ranks as the second-largest producer of hogs within the ASEAN nations [42]. Before the ASF epidemic in 2019, the country witnessed a steady expansion in its swine inventory [43]. In the year 2018, the overall hog number reached 12.60 million, reflecting a 0.83% rise from the previous year’s count [44]. In July 2019, before the ASF outbreaks, the swine inventory in the Philippines was estimated at 12.70 million heads [44]. By July 2020, this had reduced by 18.6% to 10.74 million [45], indicating high mortality and culling [46]. The most significant annual decrease of 20.8% occurred in 2021 [40], with a 41.7% (about 2.01 million units) population decrease in commercial farms [47] and a 13.30% (approximately 1.06 million units) reduction in backyard farms compared to the 2020 inventory. In 2022, the inventory stood at 9.43 million heads, representing a further 5.2% decline from the 2021 total of 9.94 million heads [40]. However, in 2023, the population had increased a little to 9.77 million [41].

**Figure 1.**
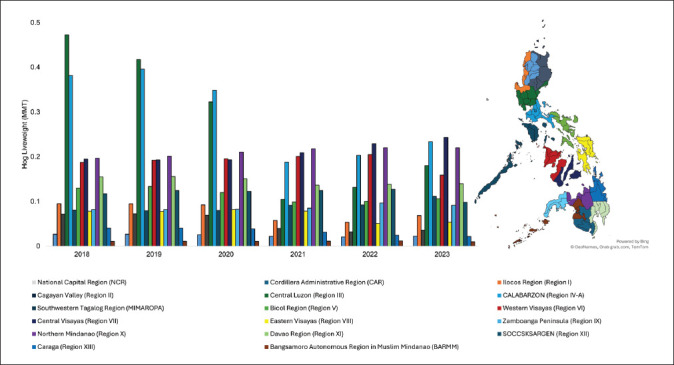
Changes in hog inventory among regions during 2018–2023.

Industrialization has led to the relocation of pig populations from rural regions to the outskirts of large urban centers, such as Manila [33]. Before the ASF outbreak, the distribution of the pig population followed a different pattern. Until 2020, Central Luzon (Region III) and CALABARZON (Region IV-A) had traditionally led in pig numbers. According to the 2021 annual swine report, primary production regions transitioned to Western Visayas and Central Visayas, representing 12.1% and 11.6% of the entire stock, respectively [40]. Western Visayas also had the highest inventory in 2022 [40]. However, Calabarzon regained the first position, followed by Western Visayas and Central Visayas in 2023 [48]. The distribution of hog inventory to 17 regions of the country is presented in Figure 1.

Despite occasional reductions in inventory, liveweight and pork production grew at average annual rates of 2.96% and 3.50%, respectively, between 1990 and 2018 [26]. From 2012 to 2019, the average yearly volume of hog production was 2.16 million metric tons (liveweight, MMT), with the highest output of 2.32 MMT recorded in 2018 [49]. The emergence of ASF in 2019 had a significant impact on hog production, resulting in a sharp decline [19,40,45,47]. Volume of production dropped to 2.14 MMT in 2020 [16], further declined to 1.70 MMT in 2021, and then slightly recovered with a 2.4% increase to 1.74 MMT in 2022 [40]. In 2023, the production volume again rose slightly to 1.79 MMT [49]. Until 2020, Central Luzon (Region III) and CALABARZON (Region IV-A) had traditionally led in hog production volume. According to the 2023 data, Central Visayas is the leading producer, followed by CALABARZON and Northern Mindanao (Region X) [41].

**Figure 2.**
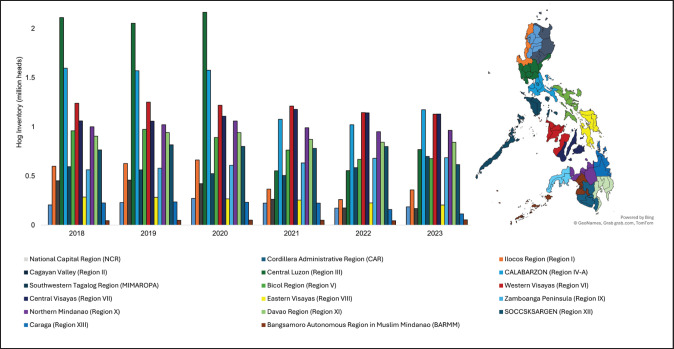
Changes in volume of hog production among regions during 2018–2023.

Compared to the third quarter of 2022, 13 regions reported an increase in hog production volume during the third quarter of 2023 [50]. However, recent local pork production has fallen short of meeting domestic demand [13]. The country’s self-sufficiency in pork has declined sharply, dropping from 87.76% in 2019 to 65.04% in 2023 [51]. The volume of hog production in all 17 regions is presented in Figure 2.

### Consumption patterns

**Figure 3.**
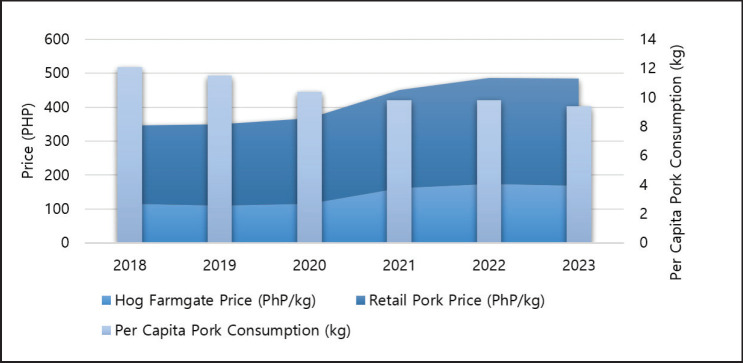
Pork price and consumption pattern during 2018–2023. (per capita pork consumption [23], hog farmgate price [55], retail pork price [56]).

Filipinos are primarily pork consumers [20], with pork consistently ranking as the most consumed meat alongside chicken [1,26]. The Philippines ranked as the tenth-largest pork consumer globally [52]. Per capita pork consumption in the country increased continuously from 2010 to 2019 [53], reaching a peak of 13.20 kg per capita in 2015 [23]. In 2019, per capita pork consumption dropped to 11.50 kg due to the ASF outbreak and the onset of the COVID-19 pandemic [54]. According to Organization for Economic Cooperation and Development (OECD) data for 2020 and 2021, consumption declined to 10.40 and 9.80 kg per capita, respectively, and further decreased to 9.40 kg in 2023 [23]. However, they also predicted a gradual increase in pork consumption in the coming years as follows: 9.80 kg, 10.10 kg, 10.50 kg, and 10.80 kg in 2024, 2025, 2026, and 2027 [23]. The trend in pork consumption over the last 6 years is presented in Figure 3.

### Rearing Systems in the Philippines

In the Philippines, two categories of swine farms are present: backyard/smallholder farms, which consist of 1–20 finishers without piglets, or 1–40 piglets, or 1–10 sows accompanied by 1–21 piglets; and commercial farms, which encompass ≥ 21 finishers, or ≥ 41 piglets, or > 10 sows with 22 piglets [26]. However, according to the revised PSA Board Resolution No. 04, series of 2022, there are now three classifications: smallholder, managing 20 sows or fewer; semi-commercial, managing 21–50 sows; and commercial, managing 51 or more sows [57]. As the data presented here are based on the years 2018–2023, we primarily chose to take the former classification system for subsequent discussion.

Pigs are significant for farmers in a backyard setting, serving as a primary source of family income, consumption, or as a form of savings [58], as well as for specialized expenses like school tuition costs [59]. The open-sided housing system is the most prevalent method [60] raising native pigs, where animals are typically nourished with an assortment of waste food or byproducts like crop residue, swill, and so on [61], leading to suboptimal nutrition, poor animal performance, and disease exposure [62–64] due to imbalanced nutrition [65,66] and weekend feed safety [67]. Some raisers utilize commercial concentrate feed for hybrid pigs [68]. Aside from feed, backyard farming, due to its small scale, has limited access to farm supplies, technological and veterinary care, loans, and market data [36,59]. They rely solely on family labor [15], lack farm records [20,26], and participate only in production with no value-added activities [69]. Aspile et al. [15] interviewed 71 hog raisers from the City of San Jose del Monte and reported the lowest selling price among the small backyard group. Another report indicated that backyard hog production in the Philippines exhibits low technical efficiency [70].

Meanwhile, commercial farms can use economies of scale by implementing advanced infrastructure and technologies in their operations [26]. This approach enables them to scale up operations, expand their market reach, and achieve higher value addition, ultimately driving increased profitability [17]. They have enhanced reproductive efficacy, characterized by a reduced incidence of mummified piglets, stillbirths, and piglet mortality before weaning [71]. In a commercial environment, piglets are weaned at a younger age, resulting in reduced fattening periods and improved technical outcomes [24]. Unlike backyard farmers, commercial swine producers play multiple roles throughout the supply chain [26]. Nieto-Pelegrín et al. [69] assert that the activities of industrial farms encompass feed processing, animal rearing, meat processing, marketing, and distribution. Commercial-scale swine companies can also achieve more net income, as larger farms incur the lowest proportion of fixed costs [15].

Backyard pig farmers in the Philippines surpass the commercial pig industry in production volume [72] and employment [73]. However, the swine industry in the Philippines is becoming increasingly commercialized [13]. As of March 2024, commercial farms and semi-commercial farms account for 27.1% and 2.4% of the swine population in the Philippines, respectively [74]. However, in certain places, the percentage is higher for commercial production systems. For example, areas with the highest pig population of Regions III and IV-A are more than 75% commercialized [24].

## Marketing and Value Chain

### Domestic market

The marketing of animals’ meat and meat products in the Philippines is fragmented, as there is typically at least one intermediary between the producer and the final processor [75]. Approximately 90% of hogs are marketed via intermediaries, resulting in price volatility [24]. From 2019 to 2023, the average farmgate price of hogs rose at an annual average rate of 12.4% [41]. In 2018, the farmgate price of hogs was PHP 115.9 per kilogram of live weight [55]. The price reduced to PHP 110.18 and PHP 115.22 in the next 2 years as an initial response to ASF and then spiked dramatically to PHP 161.31 and PHP 173.92 in 2021 and 2022, respectively [55]. In 2023, a marginal decline to PHP 168.78/kg was recorded [41]. Changes in hog farmgate prices during 2018–2023 are presented in Figure 3.

Middlemen-induced increased logistics costs negatively affect the marketing margin [76]. A lengthier supply chain results in increased costs, thereby elevating the commodity’s price [77]. A value chain analysis was performed by Ayomen et al. [78], identifying the primary participants in the Philippines as swine growers, merchants, meat retailers, meat processors, and buyers. The existence of certain intermediaries is occasionally crucial due to the absence of a structured market or auction system for smallholder farmers to engage directly [33]. Likewise, wholesalers and retailers rely on intermediaries due to challenges in locating suppliers that can fulfill their requirements [79]. Ultimately, pig producers are price takers rather than price makers [80].

The pork market in the Philippines encompasses traditional marketplaces and is significantly influenced by consumer preferences and external factors, such as disease outbreaks. Both smallholder and commercial farms mostly provide meat to neighborhood wet markets indirectly through middlemen, as Filipino consumers prefer fresh, warm, or chilled pork over frozen pork [14]. A burgeoning market for value-added goods exists, prompting producers to engage in it, as the sale of processed products yields greater profitability than that of raw meat or carcasses for all stakeholders in the supply chain [81].

### Import-export dynamics

**Figure 4.**
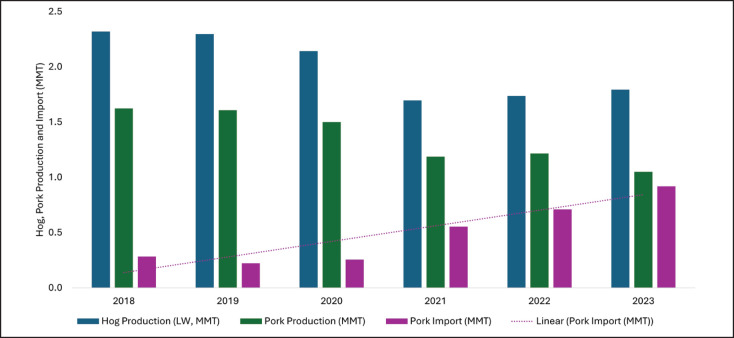
Hog and pork production and pork importation pattern during 2018–2023. (Hog production [40,41]; pork production 2018–2022 [87];, 2023 [88]; pork imports 2018–2019 [89], 2020–2023 [83]).

Pork imports in the Philippines had an inconsistent pattern impacted by factors such as disease prevalence, demand for processing-grade meat, and price changes [13]. The importation of pork has exhibited rising growth rates in recent years [51,82. From 2019 to 2023, the average annual self-sufficiency was 74.11%, whereas for 2021 to 2023, it was as low as 65.27% (calculated from [51]). The total importation in 2022 was 0.56 MMT [51], which was 252.25% higher than the 0.22 MMT imported in 2019 (calculated from [51]). In 2023, imports rose to 0.92 MMT [83], positioning it as the 19th highest importer with an expenditure of USD 481.30 million [84]. The principal sources of imported pork are Spain, Canada, the United States, the Netherlands, and France [40]. The predominant portion of pork imports was utilized in processed meat items [24] as they necessitate industrial-grade meat. In fact, 85% of the supplies required for the meat processing sector are imported [85]. President Ferdinand Marcos Jr. of the Philippines has enacted an executive order that prolongs the reduced tariff rates on imported pork for the third consecutive year, commencing in 2021, maintaining the in-quota duty at 15% and the out-of-quota rate at 25% [86]. Excessive dependence on imports will damage local producers, particularly smallholder farmers. The persistent influx of illegal pork and its byproducts is harming the local industry [24]. Pork production and importation patterns of the country during 2018–2023 are presented in Figure 4.

In 2011, the World Organization for Animal Health declared the Philippine hog industry immune to Foot-and-Mouth Disease (FMD) [43], a technical advantage over its Asian pork-producing counterparts in pork exports [14]. Nonetheless, Philippine pork remains uncompetitive in the world market due to elevated production costs, which are among the highest in Asia [24], and suboptimal production levels [13]. However, in 2022, the Philippines exported pork worth USD 0.30 million to the United Arab Emirates and Denmark, making it the 65th largest exporter [90]. The nation imposes a 30% and 40% duty on in-quota (254,210 metric tons) and out-of-quota imports, significantly exceeding Vietnam’s tariffs of 15%–25%, thereby enhancing Vietnam’s competitiveness as a pork exporter in the region [24].

### Philippine Swine Industry Challenges

#### Diseases

Maintaining optimal health is crucial for enhancing farm profitability and ensuring the well-being of pigs [91]. Agricultural illnesses often have considerable indirect and occasionally direct effects on human health and well-being, in addition to production losses [18,92]. Diseases such as ASF, FMD, and Porcine Reproductive and Respiratory Syndrome (PRRS) continue to harm the Philippine swine industry [19,93]. A high proportion of backyard farms [26], inadequate disease monitoring and control [24], and weak implementation of regulations are believed to be contributing factors to the emergence and reemergence of illnesses. FMD outbreaks in 1995 resulted in an 11.8% drop in pig farm numbers and a 15.7% decrease in wholesale prices, as documented by Abao et al. [72].

The outbreak of PRRS posed significant challenges to the local swine industry, resulting in a 2.41% annual decline in swine inventory between 2009 and 2014 [94,95]. Another notable disease outbreak, porcine epidemic diarrhea, recorded in 2005–2006, 2010, and 2014–2015, led to substantial piglet mortality [96]. A 2021 study revealed that both classical (genotype 1) and pandemic (genotype 2) strains of the porcine epidemic diarrhea virus were still present in the Philippines [97].

ASF is a hemorrhagic illness in swine, induced by the African Swine Fever Virus (ASFV) [98, 99], characterized by its high infectiousness, high mortality rate, and considerable socio-economic repercussions [43,98,100]. In Asia, it was first documented in 2018 in China and has since been disseminated to several Southeast Asian nations, including the Philippines [101–103]. The rapid outbreak during events such as the Chinese New Year [104, 105] and the Vietnamese New Year [106, 107] is believed to result from the combination of dense human and pig populations, as well as the frequent movement of people and animals. Additionally, ASFV can be disseminated through infected swine, contaminated bedding, farm surroundings, tools, leftover food, wild boar populations, fomites, and other insect vectors, both directly and indirectly [102,108–110]. Although swill feeding is considered the most common reason for outbreaks, it remains significant in Asia [95, 111]. Additionally, raw and processed pork products, whether locally sourced or imported [112], pose an elevated risk of ASF transmission, as observed in Taiwan [113] and Japan [114]. In another study, ASF antibodies were found in 3.57% and 2.27% of pigs from abattoirs in Baybay and Ormoc cities, respectively, suggesting that some cases of ASF on farms remain undetected or unreported [115].

A small backyard farm in Rizal province experienced the earliest ASF outbreak in July 2019 [116], which led to an immediate 9.8% decrease in pig production over the last 3 months of the year [103, 117, 118]. It devastated the industry so severely that numerous farmers ceased operations, and animals were exterminated and buried to curtail the propagation of the disease [18, 112]. The retrospective expenditure exceeded USD 58 million in the first year [119]. Initially, ASF led to declines in production and farmgate pricing [120], but eventually, the hog inventory dropped significantly, resulting in increased pork prices [18, 43, 112]. The situation got worse due to the added burden of the COVID-19 pandemic. Given the potential for up to 100% mortality [19, 121] and the absence of a recognized remedy, sick pigs had to be depopulated [103]. Despite control attempts, all 17 administrative regions [19, 43] and 89% (73 out of 82) of the provinces are impacted, with 5 million hogs having been culled, resulting in significant economic detriment for individual pig growers and the whole sector [122], amounting to around PHP 200 billion or more [123]. A cyclical trend in ASF events is noted from August to October (monsoon), characterized by elevated frequency and intensity, and from April to May (hot and dry), with low frequencies and severity were observed in the Philippines [103], indicating a significant relationship to precipitation [124].

ASF has spread to both small and commercial farms, as well as in feral pig herds [43, 103, 112], and may remain underreported in Asia [19, 66]. Although the chance is low [19, 67], certain regions with higher wild pig populations of domestic pigs may encounter wild boar contact [125], making the Philippines a high-risk area [66]. A study indicated that nine out of ten wild pigs exhibiting non-hemorrhagic diarrhea were carrying ASFV in Spain [69]. In May 2021, pig hunters in the Philippines documented approximately 100 wild pig fatalities in Abra Province, which yielded positive results in meat sample tests [126, 127]. In addition to wild boars, feral dogs and cats may serve as major biological carrier species [128]. In the Philippines, there is significant apprehension regarding the appropriate disposal of ASF corpses [129]. The ASFV can last in decomposing carcasses for several months, particularly in low-temperature conditions [130]. Early misinformation during the ASF epidemic prompted farmers to conceal and sell their livestock and improperly dispose of deceased animals [18, 131]. The consequences of doing so have been described in several writings [132, 133]. ASF vaccinations have been going on since 2021 [134]. Out of the initial two vaccines, one originated from a collaboration between a United States vaccine manufacturer and Zoetis, while another is the NAVET-ASFVAC from Vietnam. Both are live attenuated vaccines and reportedly failed to induce the intended response [43]. In June 2023, the Department of Agriculture (DA)-Bureau of Animal Industry recommended the AVAC ASF LIVE vaccine [135], and the government intends to commence extensive immunization by 2024 [135] even though an attenuated vaccine might not confer sterilizing protection [136].

### Biosecurity issues

The organizational framework of the swine business in the Philippines is intended to facilitate communication among herds [137]. Smallholder farms exhibit significant amounts of direct (e.g., intermingling of pigs) [33] and indirect (e.g., unauthorized access by individuals and vehicles) exposure to infectious organisms [137– 139]. Hygiene practices are typically inadequate or non-existent for interactions between farm workers and pigs outside their farms, as well as between outsiders and pigs confined on the farms [33]. Even in commercial settings, marketing vehicles and traders may come into contact with existing herds, thereby presenting a significant biosecurity hazard [139, 140]. The heightened rate of contact increases the probability and intensity of introducing pathogens, regardless of production type [138, 141].

Swine are prevalent reservoir hosts for *Salmonella* [142]. In a study, 700 *Salmonella* isolates were isolated from swine samples in abattoirs and wet markets across four districts in Metro Manila, noting that over 50% of the samples carried virulence genes [143]. Calayag et al. [2] examined 8 slaughterhouses and found *Salmonella enterica* in 44% of pigs butchered in accredited facilities and in 46.7% of pigs butchered in locally regulated slaughterhouses. These findings necessitate attention, as licensed slaughterhouses are permitted to ship meat nationally, thereby presenting a risk of a *Salmonella* epidemic to multiple cities, similar to the previously reported multi-country *Salmonella* outbreak [144]. The risk persists with processed pork. A study indicated the presence of *Salmonella* in 8% and 7% of pork-based street food samples from Taiwan and the Philippines [145]. A separate investigation revealed that all sausage samples exceeded the permissible limit for coliforms and tested positive for *Staphylococcus aureus*, while only 26.77% of the samples fell below the threshold for *Escherichia coli* [146]. Additionally, antibodies of the hepatitis E virus [147], and *Trichinella* spp*.* [148] in pork signifies a potential infection risk for people. The appearance of Reston virus in domestic swine in the Philippines has raised new concerns [149]. The presence of these risks amplifies the risk, as substantial data indicate inappropriate disposal of deceased animals [1] and on-farm slaughtering of pigs [58, 150]. Between 2018 and 2022, merely 46.87% of swine slaughtering occurred at slaughterhouses [40].

By 2050, antimicrobial resistance is projected to result in 10 million fatalities annually, predominantly in Asia [151]. Excessive use of antibiotics and antibacterials without veterinary oversight is common in the country [1, 33], with 63% of smallholders relying on agricultural supply stores for veterinary products [140]. A survey on backyard farms indicated that merely 6% of these farms acquire antimicrobials through a veterinary prescription [1]. Recent studies have recorded bacterial resistance to specific antimicrobials utilized in the Philippines [152, 153]. A high prevalence of resistance to ampicillin [2], β-lactams [143, 150], cefuroxime [150], trimethoprim/sulfamethoxazole [2], nitrofurans [2], and cefoxitin [150]; and multidrug-resistant *Salmonella* [2, 150] have been reported. This implies that pig producers do not rigorously comply with government-established veterinary policies.

#### High production costs

Significant impediments within the supply chain affecting pricing comprise a deficiency in yellow corn supplies and a scattered backyard structure [26]. Additionally, disease occurrence, the participation of intermediaries, importation, and elevated energy costs render the pricing uncertain. Commercial growers may sometimes gain from purchasing feeds and pharmaceuticals directly from firms [24], but dependence on imported genetics and technologies escalates costs [33]. A sow is potentially expected to farrow 2.35 times annually [154]. A study of 214 smallholder farms indicated an average of 1.7 L per sow annually, with an inter-farrowing interval of 211 days [20]. The research also recognized late weaning (28–45 days) and frequent sow replacement [20], neglecting the reality that a sow often does not yield a return on investment until her third litter is weaned [155]. Moreover, gilts and younger sows can diminish profitability by producing lighter, smaller litters with elevated stillborn and preweaning death rates [156]. Another notable restriction is the low technical efficiency (resource utilization efficiency) in Philippine backyard production, which is attributed to constraints in operational capital and managerial expertise [70].

Inter-island shipments in the Philippines are typically costly [157]. To circumvent inter-island transportation of hogs, it is advisable to construct additional post-production facilities in provinces with a substantial hog supply. Transporting pork instead of live animals will diminish regulatory oversight, mitigate disease transmission, and lower logistical expenses associated with transportation [24].

#### Genetics

The Philippines possesses both indigenous and non-native stocks. Indigenous breeds comprise Sus scrofa, the Philippines’ Black Tiaong, and Kalinga [28, 71]. Landrace, Large White, and Duroc are common exotic breeds, and various crossbreeds, such as Landrace × Large White, Pietrain × Large White, and Duroc × Pietrain, are also available [158, 159]. A multitude of smallholder farmers engage with indigenous sows, which are less productive than exotic sows [160, 161]. For instance, Black Tiaong and Kalinga breeds exhibit subpar farrowing and weaning performance relative to sows from commercial farms [71]. The farrowing rate of exotic sows in the Philippines’ commercial farms in 2009 was 82%, reduced to 80.44% by 2018, still falling behind neighboring pork-producing countries [24].

In 2018 and 2020, the average number of pigs sold per sow was 19.05 and 18.29 heads, respectively [24], with a target of increasing to 30 heads by 2026. The target seems too large to achieve in such a short time span, where the small scale of pig keeping is a major obstruction [33]. Breeders are predominantly obtained from neighbors and infrequently from professional breeders or commercial farms [162]. Artificial insemination (AI) is prevalent among commercial farms and is increasingly favored by small-scale producers. However, some farmers continue to favor hiring boars [24]. Several commercial stud farms (Davsaic AI Centre, Bulacan, ATI-ITCPH) offer “semen-on-demand” services, which may be prohibitively expensive for numerous raisers [24].

#### Environmental concerns

Natural resources are significantly burdened by severe pollution resulting from the profit-maximizing model of pig production [141], which is associated with soil and water contamination, odor problems, animal waste, global warming, and various other challenges [163]. A 2010 survey 2010 revealed that 23% of smallholders and 33% of commercial farms discharged effluent into water bodies, including lakes, creeks, and rivers, while 26% of smallholder and 22% of commercial farms released effluent into open spaces and rice fields [37]. Other studies have reported similar waste disposal practices [20, 141]. Such waste in surface waters reduces oxygen levels, leading to ecosystem dysfunction and a decline in biodiversity [164]. Additionally, elevated nitrous oxide emissions from nitrogen-saturated fields can lead to increased nitrate concentrations in drinking water, thereby exposing the population to environmentally associated health disorders [13, 37]. Alongside improper management of farm waste, the burial of veterinary waste contaminates the soil [37], and open-air incineration of refuse exacerbates air pollution [165] by releasing harmful substances such as dioxins, alkanes, phthalates, and polycyclic aromatic hydrocarbons [166].

Global CO_2_ emissions from livestock operations have surged by 51% over the past 50 years, driven by escalating demand for animal products [167]. In the Philippines, the livestock sector contributes nearly 10% of the total 152.34 million tons of greenhouse gas emissions [60]. Greenhouse gases subsequently diminish feed availability, propagate animal illnesses and parasites, and impair water resources [109]. GHG emissions from the feed production module are contributed to by high-carbon feed ingredients (soybean meal, yellow corn), a poor feed conversion ratio, high feed wastage, and a long distance between the feed mill and farm [60]. The importation of feed components from distant places in the Philippines exacerbates environmental degradation [168]. Moreover, the higher FCR [3.19 in 2020], which the DA planned to reduce to 2.27 by 2026 [111], and 5%–20% feed wastage in manual feeding systems [169] add to the burden. A significant portion of pork is imported from other countries, located on average at a distance of 13,959 km [84], which increases the GHG load. Greenhouse gas emissions from the animal production module account for 57.88% of intestinal fermentation and 42.32% of power consumption on the farm [60]. Commercial farms exhibit greater emissions per unit of animals due to increased mechanization and reliance on power-dependent equipment compared to open-sided housed animals. As the government’s target is to shift towards farm commercialization and mechanization, the contribution to GHG emissions is expected to increase. Alternative green energies (solar, wind, hydropower, and geothermal) ought to replace the traditional power system. An improper manure management system also contributes to GHG emissions [170]. The country has a prolonged history of inadequate animal waste management [20, 37, 141] that requires attention.

#### Feed

In the Philippines, feed expenses account for 65%–80% of costs, depending on the managerial framework employed [24, 33]. For a 90 kg marketing weight, on average, a farmer spends PHP 5,524.26 per finisher, which takes approximately 167 days, with a total feed consumption of around 217.56 kg in a commercial setup [24]. The predominant components of swine feed include yellow maize, soybeans, corn, or rice bran, among others [114]. Maize supply is limited and can significantly impact the price of pigs, as it accounts for approximately 35% of the feed cost [171]. The Philippine Association of Feed Millers, Inc. said that an annual requirement of 10 million metric tons of yellow corn necessitates imports [172], primarily from the USA, Brazil, Argentina, and other ASEAN countries. The Philippines buys more than 99% of its soybean requirements, with only 1% harvested locally [173]. Heavy dependence on imported raw materials, the COVID-19 pandemic, and the conflict between Ukraine and Russia have significantly destabilized the animal feed sector [174, 175].

#### Pork quality and price

Hog and pork providers are responsible for producing safe meat to safeguard customer health. Nevertheless, there are reports of pathogens [2, 149] and pharmaceutical residues in marketed pork [176]. Some abattoirs are alleged to maintain inadequate cleanliness standards, posing potential hazards to the population [26]. The Philippines has 132 accredited slaughterhouses, with 122 classified as class AA (local supply) and 10 as class AAA (export grade). Additionally, there are locally registered private meat enterprises producing pork for individual cities and municipalities [26]. As there are increased risks [177] and evidence [2] of pathogenic contamination in pork at the abattoir, slaughterhouses must uphold stringent cleanliness and elevated hygiene standards to avert the dissemination of diseases. At the same time, the government is obligated to thoroughly enforce the Food Safety Act (Republic Act No. 10611) [178].

Due to inadequate availability, disease outbreaks, and inflation, pork prices in the Philippines have experienced continuous fluctuations (Fig. 3). The average retail price of pork in the country has steadily increased. In 2018, the retail price of lean pork meat was roughly PHP 229.38, rising to PHP 315.63 in 2023 [56]. The escalation of pork prices in the Philippines is attributed to supply limitations, increased feed and production costs, higher transportation charges, and rising demand. Furthermore, in the contract farming model, enterprises dominate marketing, thereby rendering farmers as employees of these giant corporations [80] and hence creating a monopoly.

#### Limited technology adoption

Innovation and technology are crucial for the sustainable development of the global food system [179, 180]. A plethora of scattered tiny independent farms seems constrained in their access to current technological advancements [13]. In traditional pig farming, there is a notable absence of technical adaptation, which may be attributed to limited financial resources and a lack of enthusiasm. The Philippines’ Internet access is inferior to that of other developing nations in Asia [181], which constitutes a significant prerequisite for smart farming. For large farms, profitability increases with automation [182] as digital technologies enhance farmers’ management proficiency [183]. Several prominent commercial growers are implementing foreign-developed sensors and models on their farms. Examples of advanced facilities and technology include tunnel ventilation [14, 60]; farrowing crates [24]; automated feeders, silos, and water systems [24]; and biogas plants [184]. Some manuscripts reported the pilot use of sensor-based technology in monitoring breeding, pregnancy, and birthing processes in the country [185, 186]. The implementation of science and technology-based treatments in commercial swine farms has enhanced the production, productivity, and quality of pigs in the country. For example, tunnel-ventilated housing is considered a contributing element to the eradication of FMD [14].

Among other recent works, Baesa and Palaoag et al. [187] developed a web-based investment platform for poultry and swine farmers that enhances decision-making and optimizes investment returns by matching investors with the most suitable farms based on production data and profiles. Such approaches can be beneficial for generating credit in cooperative enterprises. Another marketing tool, called “Bugana”, is a cost-effective and secure online platform that enables farmers to sell their agricultural products directly to consumers, thereby improving market access, price transparency, and convenience [188]. The Open Academy for Philippine Agriculture evaluates and promotes promising technologies by testing various ICT models that provide timely information to farmers [189].

#### Potentials of Smart Farming

Precision livestock farming technologies have the potential to enhance animal welfare, improve feed efficiency, increase performance, and reduce livestock emissions, thereby significantly influencing the financial sustainability of pig farms [190]. A range of inventions has been introduced to improve production [191, 192]. Utilizing the internet of things (IoT) and sensors for monitoring the indoor farming environment is highly efficient [193] and convenient [194], thanks to their accuracy and reduced maintenance expenses [195]. Sensor-based systems reduce the need for continuous human oversight, as they are designed to operate around the clock [196]. The use of radiofrequency sensors in identifying and tracking individual animals [197, 198] and in feeding time computation [199]; accelerometers [200] and GPS [201] for tracking; camera systems for identification and temperature measurements [202– 205]; cameras for determining body weight and identifying injuries [191, 206– 210]; infrared cameras in estrus detection [211, 212] and fertility evaluation [213]; flow meters for measuring water intake [214]; and so on, have already been investigated by academics and companies. Artificial intelligence in smart pig farming can enhance porcine health and welfare, alleviate stress for both pigs and farmers, and augment productivity and economic efficiency [215]. Several studies using machine learning for animal recognition [216]; posture and locomotion identification [217– 219]; disease symptom detection [220]; feeding behavior analysis [197, 218, 221, 222]; body weight assessment [223, 224]; water consumption measurement [225]; and so on, are explored worldwide. The comprehensive application of sensor-based information, machine learning, and other sophisticated tools can enhance predictive accuracy and enable real-time decision-making [91, 226, 227]. Sophisticated veterinary surveillance and disease management strategies integrated with precision pig farming can facilitate the prompt identification of health concerns and the execution of timely interventions [185, 228– 230]. Research has been conducted on sensors for detecting foodborne infections in animals [231, 232], microfluidics for diagnosing ketosis [233], ZigBee-based networks for the prompt identification of *E. coli* and *Salmonella* *enteritidis* infections [234], and sound analysis for identifying respiratory diseases and stress [235– 238]. As a technology purchaser, the Philippines should prioritize selecting well-validated items as an initial step in precision farming; nonetheless, the development of indigenous products is essential for enhancing and maintaining established instruments over time.

### Feasibility of Sustainable Pig Production

#### Repopulation efforts post-ASF

The DA and National Livestock Program’s (NLP) initiated the Integrated National Swine Production Initiatives for Recovery and Expansion program in 2021, aiming to repopulate and recover the ASF-ravaged hog population [112]. The goal is to revitalize and expand the swine industry by supporting repopulation efforts, improving biosecurity measures, and promoting recovery from the impacts of ASF [162]. The DA allocated PHP 4.1 billion in 2022 for the implementation of the program [162]. Flexible loans, encouragement of public-private partnerships, and livestock insurance are expected to follow the initiative. Another initiative, known as the Bantay ASF sa Barangay, is being executed nationwide by the DA, various governmental organizations, and the corporate sector [43, 103]. Notwithstanding the obstacles presented by stakeholders, COVID-19 protocols, and labor shortages [239], the program continued to facilitate risk evaluation and inspection, enhance hygiene, and develop capabilities through education campaigns, thereby strengthening local government unit engagement and institutionalization, and promoting recovery and repopulation. Additionally, funds from the Development Bank of the Philippines and other financial institutions are provided to facilitate swine repopulation, rehabilitation, and recovery [24]. These initiatives have resulted in a 3.6% increase in the hog population [240] and 2.87% in the volume of hog production [49] between 2022 and 2023 (Fig. 4).

#### Government policies and support

The Philippines has a long history of promoting swine production. The International Training Centre on Pig Husbandry, the sole training facility in Asia and the Pacific dedicated to pig management, was established in 1985 [241]. In 2003, the Swine Breeder Farm Accreditation Program was established to discover, validate, and support pig farms with superior genetics [14]. Later, the DA-NLP initiated the AI sa Barangay project to provide smallholder farmers with superior-bred pigs [24]. Recently, understanding the low-cost involvement and ease of transportation of frozen semen, DA has planned to establish AI stations in every province to provide AI service for free [24]. The DA-NLP allotted PHP 62.5 million for the creation of 19 enhanced swine AI centers in 2022 [162].

The Philippines introduced regulations governing the sale, prescription, and distribution of antimicrobial veterinary medical products ahead of the emergence of antimicrobial resistance (AMR) as a global public health concern [242]. To address antimicrobial resistance, the application of olaquindox, carbadox, nitrofurans, chloramphenicol, and human β-agonist medications in livestock production has been prohibited [2]. In 2014, the Philippine government formed the Inter-Agency Committee on Antimicrobial Resistance, which enabled the development of the National Action Plan to Combat AMR 2015–2017, launched in May 2015 with the objective of nationwide AMR surveillance [243]. Nonetheless, the absence of implementation capacity continues to hinder progress.

For pork distribution, the government certifies abattoirs based on their infrastructure and operational protocols. Entities failing to meet these standards are monitored by local authorities and are permitted to distribute meat solely within their municipality [2]. In 1999, the Philippines implemented a risk assessment strategy for livestock imports to safeguard consumers and the livestock sector from disease-laden imports [244]. An effective zoning approach [245– 247] and mass vaccination in swine farms, livestock markets, holding yards, and abattoirs significantly contributed to addressing the endemic of FMD [43]. Subsidized vaccinations against PRRS are also provided to backyard farms [24]. Since the emergence of ASF in 2019, the government has enacted stringent measures to mitigate the disease’s spread, including national zoning, intensified surveillance, rigorous biosecurity and quarantine measures, limitations on the transportation of hogs and pork products, and the execution of diseased and at-risk animals [112]. Another initiative involved eradicating all pigs within a 1 km radius of the affected farm within 5 days, along with implementing stricter disease surveillance within a 7 km radius [248]. A nationwide state of emergency was declared for one year on 10 May 2021, acknowledging the urgency [249]. In addition, the National ASF Prevention and Control Program (BABay ASF) was initiated in 2021 to mitigate and manage ASF [43, 103]. The DA has issued an administrative order to all veterinary offices and barangay biosecurity offices to enhance monitoring and effectively enforce biosecurity protocols [112]. Checkpoints have been established to verify animal medical certificates, transport permission, and shipping company licensing [112].

The DA has identified eight Key Result Areas (KRAs) to support the hog sector’s recovery and growth [24]. To achieve the KRAs, the DA has disbursed USD 14.6 million to indemnify hog farmers impacted by ASF [24]. The establishment of the National Information Network in 1996 and the Philippine Statistics Authority in 2013 optimized the gathering and management of agricultural data.

#### Future marketing

Domestic pork demand is influenced by several factors, including price [69, 250], population growth [52, 251, 252], age group [253], changes in income [26, 69], shifts in choice [24, 26], and disease outbreaks [52, 251, 252], among others. Arcalas [254] forecasted an increase in pork production in the Philippines. By 2027, per capita pork consumption is projected to be 16.4 kg per year, requiring 13.72 million heads [255]. As of 27 November 2024, the Philippines’ population is 116.23 million and is expected to reach 129.55 million by 2040 [256]. The proportion of urban dwellers is currently 48.7% and is forecasted to reach 60.3% by 2040 [256]. In the city, people consume more meat than in rural areas [257], and young Filipinos exhibit a strong fondness for processed pork products, having developed a habit of dining out over the years [24, 26], which may potentially lead to increased pork consumption. Along with the population, the average annual income of Filipinos is also increasing; in 2020, it was USD 3,940.6, which is expected to rise to USD 5,716.6 by 2025 and to USD 8,138.1 by 2030 [258]. Furthermore, insufficient nutrients in the rice-centric diet of the Philippines [22] have prompted affluent households to transition to nutrient-rich food items, including meat and dairy products [259]. These are unequivocal markers of a nation poised for increased hog demand in the forthcoming years.

Pork offers opportunities in both the global market and domestic demand. In 2023, global pork consumption reached 122.11 MMT, with a projected increase to 131.04 MMT by 2033 [260]. In 2019, total pork imports amounted to 9.31 MMT, increasing to 9.88 MMT in 2023 [51], despite significant ASF outbreaks in China, Vietnam, and the Philippines. Due to environmental concerns, several major pork-consuming nations, including China, Japan, South Korea, France, Russia, the United States, Australia, Poland, Sweden, and the United Kingdom, are promoting the importation of pork [261– 263], which is expected to lead to a rise in pork imports. If the Philippines wants to take advantage of international trading, development in production, quality control, disease management, and marketing is essential. A good indicator is the rising quantity of animals processed at slaughterhouses, which rose annually by 3.12% from 2011 to 2020 [26].

#### Sustainable farm management practices

Strategies to enhance the sustainability of swine farming encompass quality assurance, age-segregated rearing, educational initiatives, manure management systems, phase feeding, dietary modifications, and the modernization of related businesses. The modernization of the swine sector in the Philippines is essential for the country to remain competitive both regionally and globally [15, 24]. The Philippines has set a goal to establish a profitable, viable, and world-class hog sector by 2026 [24]. In doing so, the industry must prioritize the well-being of both smallholders and the commercial production system.

Efficiency in swine production improves with scale [15]. Between 2007 and 2017, pig production in China increased by 26.6%, primarily driven by the expansion of large-scale farms [246]. In the context of the Philippines, backyard pig farmers must enhance their agricultural operations to ensure sustainability [73]. Many farmers favor hog growing and finishing production in both commercial and backyard settings. However, studies on hog economics indicate that farrow-to-finish production yields the best income [15], whereas the growing-finishing production system generates the lowest income [15]. It is imperative to shift the mindset of producers, particularly small-hold raisers, to promote selling at approximately 120 kg (19.3% return on cost), since this strategy is likely to yield greater profitability compared to selling at 80 kg (−0.7% return on cost) [24]. Regardless of the animal species and marketing age, sustainable pig farming methods, such as the deep litter system, reduce housing, labor, and water utility expenses [24]. Among the available home design options, tunnel-ventilated housing showed superior effects on daily feed consumption, body weight, and feed conversion compared to open-sided housing [264]. The Philippine Association of Meat Processors, Inc. proposed the establishment of world-class slaughterhouses [26] to yield export-quality pork. Given the increased financial investment required for constructing enhanced facilities and supplying nutritious feed, cooperative farming can incorporate multiple smallholder farms into the network [265, 266]. A business-to-business e-marketplace platform can enhance sales and profitability for small-scale swine farmers while mitigating risks and augmenting revenue for wholesalers and retailers [79]. For large-scale commercial farms, vertical integration (including feed mill, farm, processing, and marketing) has the potential to enhance profitability [267]. This technique is being adopted by some successful enterprises and cooperatives in the Philippines [24].

#### Environmental sustainability

By 2050, the worldwide population is anticipated to exceed 9.7 billion [268], necessitating an extra 110–220 million tons of meat to sustain this population [269]. This can prove to be a significant threat to the natural resources that swine farming deals with. In 2009, the OECD laid the groundwork for green growth [270] as a global initiative to address some of the world’s most pressing concerns. The geographical location of a farm substantially influences waste disposal methods and pollution levels [271]. Farms next to rivers or other open water sources are most likely to pollute the water. Certain research has indicated that larger farm sizes significantly reduce emissions into the environment [272– 274], while others have concluded that there are adverse effects [275]. Regardless of farm size, promoting climate-resilient practices to minimize water use on farms (e.g., dry cleaning initiatives, vermiculture purification) is advisable [276]. By using a modified nipple drinker (using bite ball valves in the nipple drinkers), a 15% reduction in water usage is possible [277].

Swine farms can reduce GHG emissions by using low-carbon raw materials and energy inputs, and environmentally sustainable technologies and practices that reduce waste. The Philippine Council for Agriculture, Aquatic, and Natural Resources Research and Development is studying the replacement of high-carbon soybean meal with protein-enriched copra meal in swine feed [119]. Alternative protein sources for swine feeding encompass various legumes, distillers’ dry grains, fish, and animal-derived proteins, as well as novel sources such as microalgae and insects [278]. Low-protein diets containing amino acids can also reduce the portion of soybean meal in swine feeds [279]. Mangrove palms have the potential to be a sustainable, climate-smart feed source for swine, with a 400% higher calorific yield per hectare than maize, which could potentially reduce greenhouse gas emissions and promote sustainable agriculture [280]. Many growers may have the misconception that low-carbon production systems result in lower profits. However, a study revealed that the low-carbon production system achieved a return on investment of 36.75% with an amortization period of 2.72 years, whereas the traditional system produced a return of 19.96% and required 5.01 years [60]. Improving swine manure management could reduce greenhouse gas emissions by 65% and ammonia emissions by 78% [281]. One way to achieve this is through the integrated livestock–crop production system [282], where animal excrement and agricultural byproducts are often repurposed and reused. Pig manure possesses greater levels of nitrogen [283], making it an effective fertilizer for agricultural areas and aquatic environments [284]. Therefore, integrated crop–livestock systems can enhance the environmental sustainability and profitability of farms and communities [285].

The proper use of slurries as fertilizers is another pillar for achieving sustainability in pig production [286]. Advanced manure treatment technology reduces emissions [287] and produces nutrient-rich bio-solids, fiber, and reclaimed water [288]. Varma et al. [289] employed swine manure treatment systems and identified beneficial effects on the surrounding ecosystem. Kunz et al. [290] commented that phyco-remediation using microalgae can effectively treat swine wastewater, removing nitrogen, phosphorus, and metal ions, while generating valuable by-products and services for the circular economy. Another study found that optimizing swine wastewater treatment with a 12-day hydraulic retention time can produce high-value microalgae biomass, which can be used to supplement swine feed and provide clarified water for pig stalls [291]. Silveira et al. [292] compared geofiltration of swine farm wastewater with vermifiltration and suggested that vermifiltration using vermiwash can enhance wastewater treatment performance, reducing copper dispersion and improving nutrient recovery and water resource recycling. Mungruaiklang and Iwai [293] asserted that decomposition, anaerobic breakdown, and the pyrolysis of manure into biochar are efficient methods for reclaiming energy. Slurry separation and anaerobic digestion are promising methods for manure treatment, according to Dróżdż et al. [294]. Hou [295] proposed that on-farm excreta segregation and denitrification of the fluid component are viable choices for sustainable manure treatment. Numerous studies have corroborated slurry separation as an effective method for reducing greenhouse gas emissions and producing eco-friendly fertilizer [296,297]. Waste treatment facilities to convert biogas into electricity have been installed in some breeder farms, with most of them enrolled in the “Clean Development Mechanism” program [14]. Numerous industrial hog farms in the Philippines have implemented the Covered Inground Aerobic Reactor to produce power from biogas [14].

## Understanding The Recent Trend

**Figure 5.**
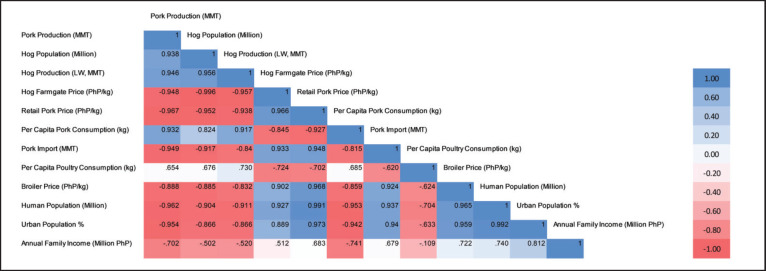
Correlation matrix of pork demand, production, and consumption-related parameters during 2018–2023. (LW: live weight; per capita pork consumption [23]; hog farmgate price [55]; retail pork price [56]; hog production [40,41]; pork production 2018–2022 [87], 2023 [88]; per capita poultry consumption [23]; broiler price [298]; pork import 2018–2019 [89], 2020–2023 [83]; annual family income 2018 [299], 2019–2023 [300]; hog population [249]; human population [301]; urban population [301]).

The correlation matrix illustrates the relationships between pork production and various other factors in the Philippines during 2018–2023 (Fig. 5). The hog population, the live weight of hogs, and per capita pork consumption are highly positively correlated to the nation’s pork production (*r* = 0.938, 0.946, and 0.932, respectively). Highly negative correlations were observed between pork production and farmgate price, retail price, and pork importation (*r* = −0.948, −0.967, and −0.949, respectively). The negative correlation between pork production and human population, urban population, and annual family income is not typical but may have resulted from the impact of ASF.

## Recommendations and Conclusion

Continuous strikes from a series of diseases have significantly hindered the Philippines’ swine industry’s progress. Right now, the primary goal should be focused on achieving self-sufficiency and producing safe pork. Improving the Philippine swine industry requires a strategic approach that focuses on farmer education, cooperative upscaling, and enhanced practices, such as early weaning (≤28 days), to shorten production cycles and extend breeding sows to at least four farrowings for improved profitability. Ensuring an adequate water supply, adopting efficient feeding systems, and utilizing low-carbon feed alternatives can all contribute to enhanced sustainability. Strengthening artificial insemination support, implementing waste management programs such as biogas production, and enforcing strict trader registration and drug prescription regulations are crucial. Upgrading abattoirs, establishing a pork traceability system, and enhancing surveillance during monsoon outbreaks of ASF can improve biosecurity and foster consumer trust. Promoting modern technologies, alternative energy sources, and livestock insurance will support long-term resilience. Meeting all these criteria will provide a significant challenge for the country. Adopting technical breakthroughs and governmental support through incentives and policies that encourage the use of smart farming technologies and sustainable practices should be implemented.

## Listofabbreviations

AA, Local supply grade slaughterhouse; AAA, Export grade slaughterhouse; AI, Artificial insemination; AMR, antimicrobial resistance; ASF, African Swine Fever; ASFV, African Swine Fever Virus; CO^2^, Carbon dioxide; DA, Department of Agriculture; DA-NLP, Department of Agriculture - National Livestock Program FMD, Foot-and-Mouth Disease; GHG, Greenhouse gases; GPS, Global Positioning System; KRAs, Key Result Areas; LW, live weight; MMT, million metric ton; NLP, National Livestock Program; PHP, Philippine pesos; PSA, Philippine Statistics Authority; PRRS, Porcine Reproductive and Respiratory Syndrome; OECD, Organization for Economic Cooperation and Development.
